# Effects of Different Levels of Coriander (*Coriandrum sativum*) Seed Powder and Extract on Serum Biochemical Parameters, Microbiota, and Immunity in Broiler Chicks

**DOI:** 10.1155/2014/628979

**Published:** 2014-12-28

**Authors:** Hesam Hosseinzadeh, Ali Ahmad Alaw Qotbi, Alireza Seidavi, David Norris, David Brown

**Affiliations:** ^1^Department of Animal Science, Islamic Azad University, Rasht Branch, P.O. Box 41857-43999, Rasht, Iran; ^2^Department of Agricultural Economics and Animal Production, University of Limpopo, Private Bag X1106, Sovenga 0727, South Africa

## Abstract

The use of herbs and spices has gained increasing interest as feed additives and possible alternative to antibiotics in poultry production. The effects of using different levels of coriander seed powder or extract on selected blood parameters, intestinal microflora, and immune response of broiler chickens were investigated in this study. A total of 420-day-old broiler chicks were randomly assigned to 7 treatments with 4 replicates and fed for 42 days. Results showed that inclusion of 2.0% coriander powder in broiler diets lowered total cholesterol while blood urea was significantly higher in birds on T_4_ compared to T_1_ and T_2_. Furthermore, there were no treatment effects on *Lactobacillus* bacteria; however, the population of *E. coli* was significantly higher in the ileum of chickens fed T_0_. Noticeable significant improvements of antibody titer against Newcastle, infectious bronchitis, and infectious bursal disease were observed in birds receiving coriander extract in water. Immunoglobulin G antibody against sheep red blood cells showed significant improvement in birds fed T_3_; likewise, immunoglobulin M was significantly higher in birds on T_2_ and T_3_ at 28 d of age. These results revealed that coriander extract or powder can be used as antibiotic alternative in broiler feeds.

## 1. Introduction

Over the years, antibiotics have been traditionally administered to poultry in order to prevent pathogenic microorganism, thereby increasing some useful microorganism in the intestinal microflora. The presence of these microorganisms in the gut is imperative for effective immunological, physiological, nutritional, and protective health in poultry [1]. Additionally, blood parameters have been shown to be major indices of physiological, pathological, and nutritional status of an organism and changes in the constituent compounds of blood when compared with normal values could be used to interpret the metabolic stage of an animal as well as quality of feed [2]. Recently, the use of in-feed antibiotics has been banned in many countries due to their residual effects in the animal and the development of antibiotic-resistant bacteria. The search for alternatives has gained increasing interest in animal nutrition in recent years. Consequently, several authors have investigated alternatives to antibiotics [3–5]. Herbs and spices have recently emerged as alternatives to antibiotics in animal production. They are known to exert antimicrobial actions* in vitro* against important pathogens, including fungi [6]. It has also been reported that plant extracts and spices as single compounds or as mixed preparations can play a role in supporting both performance and health status of the animal [7]. Guo et al. [8] observed in their study that plant extracts led to a decrease in the numbers of harmful bacteria in the caecum while at the same time increasing the numbers of beneficial bacteria.

Coriander (*Coriandrum sativum*) is considered both as an herb and as a spice. It has also been used as a medicine for thousands of years. As a medicinal plant, coriander has been used as an antifungal [9], antioxidant [10], hypolipidemic [11], antimicrobial [12], hypocholesterolemic [13], and anticonvulsant substance [14]. The major compounds present in essential oil are linalool (67.70%); *α*-pinene (10.5%); *γ*-terpinene (9.0%); geranyl acetate (4.0%); camphor (3.0%); and geraniol (1.9%) [15] (Table 1).

However,* in vivo* reports on the effect of coriander seed or extracts on selected blood parameters, intestinal microflora, and immunity in poultry are limited and inconclusive. Furthermore, the mode of action of coriander in poultry has not been fully clarified yet. Therefore, the aim of this present study was to examine the effects of using different levels of coriander seed powder or extract as a diet ingredient on selected blood parameters, intestinal microflora, and immune response in broiler chickens.

## 2. Materials and Methods

A feeding trial was conducted at the poultry farm of the Animal Science Department, Rasht Branch, Islamic Azad University. The study was carried out in an environmentally controlled poultry house. Birds were housed in pens. Floor pens were assigned to the 7 treatments. The distribution of experimental pens was arranged so as to avoid any effects of location in the poultry house. A total of 420-day-old broiler chicks (Ross 308) were randomly allocated into 7 treatments of 60 birds per group with four replications per treatment.

### 2.1. Management of the Birds

The birds had free access to feed and water. Each pen was equipped with a feeder and drinker. New wood shavings were used as litter. The initial brooding temperature was held at 32°C for the first three days and then gradually lowered to 23°C by the end of the experiment. Photoperiods were maintained at 24 h/d during the first week and decreased to 23 h/d for the rest of the trial. The birds were vaccinated against infectious bronchitis disease (IB) on the 1st and 7th day of age (infectious bronchitis virus H120; Razi Co, Karaj Iran), Newcastle disease (ND) on the 1st and 7th day of age (Newcastle lentogenic vaccine: strains Hitchner B1 and Lasota; Razi Co, Karaj, Iran), avian influenza (AI) on the 1st day of age (avian influenza-H9N2; Razi Co, Karaj, Iran), and infectious bursal disease (IBD) aka Gumboro disease on the 21st day of age (Gumboro IBD0711R; Razi Co, Karaj, Iran). No mortality was recorded throughout the experimental period.

### 2.2. Production Process of Coriander Seeds

Coriander crop requires a cool climate during the growth stage and warm dry climate at maturity. It can be cultivated in most types of soils, but well-drained loamy soil suits the crop well. Cold climate and high altitudes may lead to superior quality seed and higher essential oil content. Crop duration of coriander is about 110–140 days. Harvested plants are dried in the sunlight for 1-2 days to bring the moisture levels down to 18%. This dried plant is then thrashed to remove the seeds. Seeds are further dried in the shade to bring the moisture levels down to 9%. Coriander seed is mainly processed into powder by crushing, and this powder, with its aroma, is used as a food ingredient. The seeds are also used to extract essential oils.

### 2.3. Preparation of Aqueous Extract of Coriander

Dried coriander seeds were homogenized to a fine powder. For coriander extract, the seeds were washed well with water, air-dried at room temperature, and then ground in an electric grinder to have a coarse powder. Fifty grams of the seed powder was suspended in distilled water (500 mL) and heated to boil under reflux for 30 min. The decoction obtained was centrifuged, filtered, frozen at −20°C, and lyophilized (FreeZone Dry 4.5, USA) to give a residue (yield = 10% w/w). For assuring stability, the residue was stored at −20°C until used, and* Coriandrum sativum* extract was prepared in distilled water on the day of experiment. The proximate analysis of coriander powder was determined according to AOAC [16], as indicated in Table 2.

### 2.4. Treatments and Feeds

Seven treatments were used in this experiment as shown below:

 treatment 1: a control diet without coriander (T_0_);
 treatment 2: a control diet without coriander +750 ppm coriander extract in drinking water from 1st to 42nd days of age (T_1_);
 treatment 3: a control diet without coriander +1000 ppm coriander extract in drinking water from 1st to 42nd days of age (T_2_);
 treatment 4: a control diet without coriander +1250 ppm coriander extract in drinking water from 1st to 42nd days of age (T_3_);
 treatment 5: control diet with 1.5% coriander powder from 1st to 42nd days of age (T_4_);
 treatment 6: control diet with 2.0% coriander powder from 1st to 42nd days of age (T_5_);
 treatment 7: control diet with 2.5% coriander powder from 1st to 42nd days of age (T_6_).

The experimental diets were formulated according to NRC [17]. The ingredients and chemical compositions of the experimental diets are presented in Tables 3 and 4.

### 2.5. Serum Collection

Blood samples were collected from the brachial vein at 42 d of age from 12 birds per treatment group (3 birds per replicate). Sera were harvested from clotted blood by centrifugation at 2000 g for 15 min. Serums were analyzed for glucose, total cholesterol, triglycerides, alkaline phosphatase, uric acid, high-density lipoprotein (HDL), and low-density lipoprotein (LDL) by using the specific kits (Pars Azmoon, Tehran, 2009) and a spectrophotometer (UV) at 546 nm wavelength. The assay was carried out according to the manufacturer's protocols.

### 2.6. Immunological Traits

For immunity assays, three birds from each replicate were randomly selected and blood samples were taken on the 1st, 35th, and 42nd d of age for immunological analyses against ND and AI. Similarly, blood samples were taken on 28th and 42nd days of age for immunological analyses against IB. Blood samples were also taken on 21st and 42nd days of age for immunological analyses against IBD/Gumboro. Antibody titer was assayed by enzyme linked immunosorbent (ELISA) for IB and IBD; haemagglutination inhibitions (HI) test for ND and AI as described by Thayer and Beard [18].

### 2.7. SRBC Response

Two male birds were selected from each of the replicated groups at 28 days of age and were immunized intramuscularly with 0.2 mL of a 10% suspension of SRBC. On 35 days of age (7 days after primary immunization), blood samples were collected from the brachial vein and previously immunized birds were again injected with the same dose of SRBC. Blood for secondary antibody titers was collected 7 days after secondary immunization (on 42 days of age). The antibody titer against SRBC was determined by haemagglutination (HA) test and expressed as log⁡^2^ values for the reciprocal of the highest titer where complete agglutination was observed [19]. To measure anti-SRBC immunoglobulin G and M (IgG and IgM) antibodies, serum samples were treated with 0.2 M 2-Mercaptoethanol (2-ME) for 30 min at 37°C. This treatment inactivates IgM, and, as a result, hemagglutination observed after treatment with 2-ME is due to the presence of IgG antibodies. The difference between total antibody and IgG titers determines the IgM titer. The reciprocal of the highest dilution with complete agglutination was taken to represent titer.

### 2.8. Microbiological Traits

At 21 and 42 d of age, 16 birds from each treatment were euthanized by cervical dislocation. Small intestines were immediately eviscerated and ileum contents were gently removed into sterile sampling tubes and immediately transferred on ice to the laboratory for microbiological examination. The samples were placed in sterile glass petri dishes and were immediately refrigerated.* Escherichia coli* were cultured on MacConkey agar (Merck, Germany) at 37°C for 24 hours, and the presence of* E. coli* was determined. Examination of* Lactobacilli* was performed on tomato juice agar, after 48 h incubation at 37°C under anaerobic conditions.

### 2.9. Statistical Analysis

The data were analyzed by one-way analysis of variance SAS [20]. The completely randomized design (CRD) model included 7 treatments containing coriander powder and extract as main effects. Where there were significant differences (*P* < 0.05), Duncan's multiple range test was used to test the significance of differences between means. The model used was Yij = *μ* + Ai + eijk, where *μ* = the common mean, Ai = the effects of the coriander, and eijk = the effect of error. Before performing the statistical analysis of data, all data were tested by normality test.

### 2.10. Ethic Rules

Use and care of birds in this study were approved by the Islamic Azad University Ethics Committee. The experimental procedure described herein was also approved by this committee, and care was taken to minimize the number of birds used.

## 3. Results and Discussion

### 3.1. Performance Indices

Effects of different dietary levels of coriander seed powder or extract on weight gain of Ross 308 broilers at 42 d of age are presented in Table 5.

There were no treatment effects (*P* > 0.05) in weight gain from d 1 to 21. Similarly, from d 22 to 42, there were no differences (*P* > 0.05) in weight gain between treatments; however, weight gain was higher (*P* < 0.05) than that of the control group. During the total period, weight gain was also similar (*P* > 0.05) between treatments, but higher (*P* < 0.05) than that of the control groups except for the 750 and 1250 ppm coriander extract in water, where the weight gain did not differ (*P* > 0.05) from that of the control. Previous studies have reported the beneficial effects of coriander on broiler performance. [21–23]. This positive improvement could be attributed to the essential oil, linalool, present in coriander seed. It has been documented that coriander oil possesses antimicrobial properties against selected pathogenic and saprophytic microorganisms [24, 25]. The inhibition of harmful intestinal microorganisms by coriander oil will have positive effect on poultry health and performance as seen in this study. Çabuk et al. [26] likewise reported that linalool affected* Escherichia coli*,* Pseudomonas aeruginosa*,* Salmonella typhimurium*,* Staphylococcus aureus*,* Clostridium botulinum,* and* Clostridium perfringens* negatively.

Effects of different dietary levels of coriander seed powder or extract on feed conversion ratio (g feed/g gain) of Ross 308 at 42 d of age are presented in Table 6.

There were no treatment effects (*P* > 0.05) in FCR during the starter period. However, birds fed coriander powder in diet or extract in water had better FCR (*P* < 0.05) than the control group during the grower period. FCR was not improved (*P* > 0.05) in the control group, 1000 ppm and 1250 ppm coriander extract in water as compared to other treatments during the total period (T_0_, T_2_, and T_3_, resp.). The improved FCR obtained with coriander treatments further confirmed the benefit of this herb. The improvement in feed conversion can also be attributed to the active component (linalool) found in coriander, causing greater efficiency in the utilization of feed, resulting in enhanced growth. Additionally, Przybilla and Weiss [27] reported that the mode of action of the herb mixtures on feed conversion is through the enhancement of the digestive functions. Rajeshwari and Andallu [28] reviewed that coriander essential oil is an excellent appetizer and helps in proper secretion of enzymes and digestive juices in the stomach, thus stimulating digestion and peristaltic motion which in turn improve FCR.

### 3.2. Serum Parameters

Effects of different dietary levels of coriander seed powder or extract on hematological parameters of Ross 308 broilers at 42 d of age are presented in Table 7. Glucose concentration was significantly higher (*P* < 0.05) in the control group (T_0_) as compared to the birds receiving coriander extract in water (T_1_, T_2_, and T_3_). However, there were no significant differences (*P* > 0.05) in glucose concentration in birds receiving coriander powder and the control group. In this study, coriander extract in water decreased the glucose concentration below that of the control group. Al-Jaff [23] reported that coriander seed at a level of 1% numerically lower serum glucose as compared to the birds in the control group. His result was supported by Al-Mashhadani et al. [29] who reported that the inclusion of coriander oil at levels between 0.5% and 1% resulted in a significant decrease in serum glucose in broilers raised during summer months. The antihyperglycemic action of coriander has been documented [28]. These authors [28] reviewed that coriander incorporated into the diets (62.5 g/kg) and drinking water (2.5 g/L, prepared by 15 min decoction) reduced hyperglycemia of streptozotocin-diabetic mice. The antihyperglycemic action of coriander is associated with stimulation of insulin secretion and enhancement of glucose uptake and metabolism by muscle, reflecting the effects of more than one active constituent [28]. In the present study, the antihyperglycemic activity of coriander was noticeable in birds that were given coriander extract in water.

Birds on treatment T_5_, that is, 2.0% coriander in diet, had lower (*P* < 0.05) total cholesterol compared to the other groups. Al-Jaff. [23] reported that the inclusion of coriander seed at 2% level significantly decreases serum cholesterol by about 8.27%. Research has suggested that herbs can be effective in reducing the risk of heart attack and stroke by lowering total and LDL cholesterol and triacylglycerol concentrations without affecting HDL-cholesterol concentration [30, 31]. Dhanapakiam et al. [32] reported that rats fed powder coriander seed had a significant decrease in cholesterol compared to the control group. This report is consistent with that of Joshi et al. [33]. Our results showed that the administration of coriander seed powder had a profound influence on the metabolism of lipids in animals fed on cholesterol containing diets. According to Dhanapakiam et al. [32], the key enzyme in the pathway of cholesterol biosynthesis in the liver is 3-hydroxy-3-methylglutaryl coenzyme A (HMG CoA) reductase, a microsomal enzyme. The activity of this enzyme significantly increased in animals fed coriander seed powder as compared to the control group. Lowering of cholesterol levels in serum seems to be mediated through their increased rate of degradation to bile acids and neutral sterols. In the present study, addition of coriander seed powder in the diet of broiler chickens, that is, 2.0% inclusion level, showed significant hypolipidemic effects of coriander.

Blood uric acid was significantly higher (*P* < 0.05) in birds on treatment T_4_ compared to T_1_ and T_2_. Iyayi and Tewe [34] reported that serum urea depends on both the quality and quantity of the protein supplied in the diet. Higher level of urea in this study could be attributed to the presence of some antinutritional factors which might have lowered the quality of the protein indicating imbalance of amino acids in the diet which caused blood elevated blood urea concentration [35]. It has been documented that coriander contains active phenolic acid compounds, including caffeic and chlorogenic acid [28]. The flavonoids include quercetin, kaempferol, rhamnetin, and apigenin. Most of these compounds are known to inhibit free radicals generated in the cellular system, when they are obtained through the diet. There is limited understanding of the mechanisms through which they act. It should also be noted that kidney malfunction may also raise the level of blood urea [36].

There were no treatment effects (*P* > 0.05) on triglycerides, alkaline phosphatase, and HDL and LDL cholesterols. Our results are consistent with the report of Mansoub [37]. Al-Jaff, [23] reported that alkaline phosphatase and serum cholesterol LDL showed a significant decrease in chicks fed 2% coriander seed while HDL increased significantly in birds fed 2% and 3% coriander seed, respectively. The discrepancy observed in this study is not clear. However, Brenes and Roura [38] reviewed that harvesting time and state of maturity of plants, extraction methods of plants, methods and duration of conservation and storing, and possible synergistic or antagonistic effect of the bioactive compounds are other factors that could affect the results of* in vivo* experiments. Furthermore, the variance found in these results could be attributed to different experimental designs and amount of diet incorporated in the investigation. More research is needed to clarify this result.

### 3.3. Microbiological Traits

Effects of different dietary levels of coriander seed powder or extract on ileum microflora parameters of Ross 308 broilers from 1 to 6 weeks of age are presented in Table 8. There was no treatment effect (*P* > 0.05) on* Lactobacillus* bacteria at 21 and 42 d of age. This is in agreement with the report of Akbarian et al. [39]. Vidanarachchi et al. [40] observed that the numbers of lactose-negative enterobacteria and* Bifidobacteria* in the ileum and caeca were unaffected by dietary inclusion of the plant extracts.* Lactobacilli* and* Bifidobacteria* are considered beneficial bacteria that contribute to a balanced gut microflora and may provide an optimal precondition for effective protection against pathogenic microorganisms and an intact immune system [40]. According to Si et al. [41], it is possible to select plant bioactive compounds with a strong antimicrobial action against gut pathogens whilst not harming beneficial bacteria such as* Lactobacillus* and* Bifidobacteria*. It should be noted in this present study that the population of* Lactobacillus* bacteria considered to be beneficial in the gut microflora was numerically lowered in chickens fed the control diet at 21 and 42 d of age.

The population of* Escherichia coli *(*E. coli*) in the ileum was higher (*P* < 0.05) in chickens fed the control diet (T_0_) at 21 and 42 d of age as compared to the rest of the groups, except T_6_. Guo et al. [8] reported that plant extracts led to a decrease in the numbers of harmful bacteria (*E. coli*) while at the same time increasing the numbers of beneficial bacteria (*Lactobacilli* and* Bifidobacteria*). This finding is in agreement with our result. Rajeshwari and Andallu [28] reviewed that formulation of horsemint-fennel-coriander was found effective against gram-positive microorganisms. Antimicrobial activity and gut microbiota modulation of coriander are attributed to the essential oils present in the plant [42, 43]. The hydrophobicity of these essential oils enables them to partition lipids in the bacterial cell wall, disturbing the structures and rendering them more permeable, thus leading to the death of harmful bacteria [44].

### 3.4. Immune Responses

Effects of different dietary levels of coriander seed powder or extract on immunological responses against ND and AI of Ross 308 broilers from 1 to 6 weeks of age are presented in Table 9. There was no treatment effect (*P* > 0.05) on antibody titer against ND on 1 and 35 d of age, respectively. However, noticeable significant improvement (*P* < 0.05) of antibody titer against ND was observed on 42 d of age in birds on T_1_, T_2_, and T_3_. Al-Jaff [23] reported noticeable enhancement of antibody titer against ND when birds were supplemented with 2% coriander seed in diet. The noticeable improvement of antibody titer against ND in this present study might be due to the effects of active components in coriander oil. It has been postulated that coriander essential oil contains linalool 67.7 percent, *α*-pinene 10.5 percent, *γ*-terpinene 9.0 percent, geranyl acetate 4.0 percent, camphor 3.0 percent, and geraniol 1.9 percent [15].

Antibody response to AI was unaffected (*P* > 0.05) by treatments on 1, 35, and 42 d of age, respectively. In this current study, coriander treatments failed to induce any significant impact on antibody titers against AI. This nonsignificant effect may be due to strict biosecurity measures observed throughout the experimental period. However, further investigation is needed to clarify this observation.

Data from Table 10 showed that antibody titer against IB was similar (*P* > 0.05) for birds in T_0_, T_2_, T_3_, and T_6_ at 28 d of age. Similarly, antibody titers were significantly higher (*P* < 0.05) on 42 d of age for birds in T_2_ and T_3_. Antibody response to IBD on 21 d was significantly higher (*P* < 0.05) in birds on T_1_, T_2_, and T_3_. However, there were no significant differences (*P* > 0.05) in antibody titer against IBD in birds on coriander treatments and the control groups at 42 d of age, except T_6_. The significant improvement in antibodies titers against IB and IBD might be attributed to the activity of coriander essential oil. These activities include membrane disruption by terpenoids and phenolics, metal chelation by phenols and flavonoids, and effect on genetic material by coumarin and alkaloids that are thought to inhibit growth of microorganisms [45].

Total antibody response to SRBC was similar (*P* > 0.05) in birds on T_0_ and T_3_ on 28 d of age as seen in Table 11. Similarly, immunoglobulin G antibody against SRBC on 28 and 42 d of age was significantly higher (*P* < 0.05) in birds on T_3_. Immunoglobulin M antibody against SRBC was significantly higher (*P* < 0.05) in birds on T_2_ and T_3_ at 28 d of age, but, on the 42 d of age, the response (*P* > 0.05) was similar in all the treatments except T_1_ that was lower than the control group. Significant response of coriander treatments to SRBC was expected because this spice increased the stimulation of nonspecific immune system. Cherng et al. [46] reviewed that the aqueous crude extract in coriander has been shown to stimulate the peripheral blood mononuclear cells (PBMCs) and increase INF-*γ* secretion. These PBMCs are critical component in the immune system to fight infection and adapt to intruders [47]. Furthermore, Cook and Samman [48] noted that herbal extracts stimulate immune response by increasing vitamin C activity.

Nimmerjahn and Ravetch [49] reported that IgG plays a crucial role in the clearance of invading microbes and the generation of long-lasting immunity. The mechanism by which coriander led to an increase in IgG and IgM is unknown; however, Sproul et al. [50] reported that the B-lymphocytes synthesize specific antibodies called immunoglobulins which deal with foreign pathogens.

## 4. Conclusion

The findings of this study showed that coriander improved performance indices and ileum microflora in broiler chicks. It also induces immune response in the studied population. Reduction in pathogenic bacteria in the digestive tract will improve the intestinal health and general well-being of the birds. It is concluded that coriander powder or extract may potentially be used as an antibiotic alternative in poultry production.

## Figures and Tables

**Table 1:** Chemical composition of the essential oil from *Coriandrum sativum* seeds.

Compound^a^	Composition (%)
Heptanol	0.78
α-Thujene	0.19
α-Pinene	10.5
Sabinene	0.58
β-Pinene	0.14
α-Terpinene	0.39
p-Cymene	2.52
Limonene	0.62
Camphor	3.0
Linalool	67.70
Geraniol	1.9
γ-Terpinene	9.0
Menthol	0.54
Geranyl acetate	4.0
Borneol	1.27
Nerol	1.98
Eugenol	0.76
β-Caryophyllene	3.26
Carvacrol	0.29
l^3^-Carene	0.11
1,8-Cineol	0.97
(z)-β-Ocimene	0.41
Terpinene-4-ol	0.14
p-Cymen-8-ol	0.29
α-Terpineol	0.18
*cis*-Dihydrocarvone	0.35
Nerol	1.98
Neral	0.29
Carvone	0.24

^a^Identification based on authentic standards, NIST library spectra, and literature. Source: Khani and Rahdari [51] and Nadeem et al. [15].

**Table 2:** Nutritional composition of coriander seed powder.

Component	Coriander seed (%)
Dry matter	88.0
Crude protein	15.27
Crude fiber	33.64
Ether extract	20.0
Ash	9.50

**Table 3:** Feed ingredients of diets during the starter (1st–21st days of age) and grower (22nd–42nd days of age) periods.

Ingredient (g/kg)	Starter				Grower			
	Treatment				Treatment			
Coriander seed powder	0	15.0	20.0	25.0	0	15.0	20.0	25.0

Wheat bran	25.0	10.0	5.0	0.0	25.0	10.0	5.0	0.0
Corn	504.5	504.5	504.5	504.5	551.3	551.3	551.3	551.3
Soybean meal	393.5	393.5	393.5	393.5	316.5	316.5	316.5	316.5
Soybean oil	31.8	31.8	31.8	31.8	66.0	66.0	66.0	66.0
Ca (22%) P (18%)	21.1	21.1	21.1	21.1	17.3	17.3	17.3	17.3
CaCO_3_	9.6	9.6	9.6	9.6	8.6	8.6	8.6	8.6
NaCl	2.7	2.7	2.7	2.7	2.8	2.8	2.8	2.8
DL-Methionine	2.4	2.4	2.4	2.4	2.6	2.6	2.6	2.6
L-Lysine-hydro-chloride	0.8	0.8	0.8	0.8	0.6	0.6	0.6	0.6
Threonine	0.7	0.7	0.7	0.7	0.9	0.9	0.9	0.9
Sodium bicarbonate (NaHCO_3_)	2.8	2.8	2.8	2.8	2.7	2.7	2.7	2.7
Vitamin and mineral premix^*^	5.0	5.0	5.0	5.0	5.0	5.0	5.0	5.0

Total	1000	1000	1000	1000	1000	1000	1000	1000

^*^Calcium pantothenate: 4 mg/g; niacin: 15 mg/g; vitamin B6: 13 mg/g; Cu: 3 mg/g; Zn: 15 mg/g; Mn: 20 mg/g; Fe: 10 mg/g; K: 0.3 mg/g; vitamin A: 5000 IU/g; vitamin D3: 500 IU/g; vitamin E: 3 mg/g; vitamin K3: 1.5 mg/g; vitamin B2: 1 mg/g.

**Table 4:** Nutrient analysis of diets during the starter (1st–21st days of age) and grower (22nd–42nd days of age) periods.

Calculated nutrients								
Energy (ME) (kcal/kg)	3019.8	3019.8	3019.8	3019.8	2995.0	2995.0	2995.0	2995.0
Crude protein (%)	20.48	20.48	20.48	20.48	19.39	19.39	19.39	19.39
Calcium (%)	1.00	1.00	1.00	1.00	0.85	0.85	0.85	0.85
Available phosphorus (%)	0.50	0.50	0.50	0.50	0.42	0.42	0.42	0.42
DCAB (mEq/kg)	236	236	236	236	202	202	202	202
Lysine (SID^**^) (%)	1.15	1.15	1.15	1.15	0.96	0.96	0.96	0.96
Methionine (SID) (%)	0.50	0.50	0.50	0.50	0.48	0.48	0.48	0.48
Methionine + cysteine (SID) (%)	0.83	0.83	0.83	0.83	0.78	0.78	0.78	0.78
Threonine (SID)^**^ (%)	0.79	0.79	0.79	0.79	0.71	0.71	0.71	0.71

^**^SID (standardized ileal digestible).

**Table 5:** Weight gain mean (±SEM) of Ross 308 broilers at starter (1–21 days), grower (22–42 days), and total periods (1–42 days) fed diets or water containing the different levels of coriander (*Coriandrum sativum* L.) powder and extract (g/bird/day).

Treatment	Trait		
	Starter period	Grower period	Total period
0 (control)	38.125^a^	73.363^b^	55.744^b^
750 ppm coriander extract in water	35.946^a^	83.226^a^	59.586^ab^
1000 ppm coriander extract in water	38.155^a^	83.060^a^	60.607^a^
1250 ppm coriander extract in water	37.756^a^	82.577^a^	60.167^ab^
1.5% coriander powder in diet	40.006^a^	84.161^a^	62.083^a^
2.0% coriander powder in diet	38.821^a^	84.744^a^	61.783^a^
2.5% coriander powder in diet	39.054^a^	81.821^a^	60.438^a^
SEM	1.009	2.560	1.447

^a,b^Means (± standard error) within each column of dietary treatments with no common superscript differ significantly at *P* < 0.05.

**Table 6:** Feed conversion ratio mean (±SEM) of Ross 308 broilers at starter (1–21 days), grower (22–42 days), and total periods (1–42 days) fed diets or water containing the different levels of coriander (*Coriandrum sativum* L.) powder and extract.

Treatment	Trait		
	Starter period	Grower period	Total period
0 (control)	1.307	2.109^c^	1.838^c^
750 ppm coriander extract in water	1.372	1.830^a^	1.693^ab^
1000 ppm coriander extract in water	1.369	1.982^b^	1.792^c^
1250 ppm coriander extract in water	1.364	1.940^ab^	1.761^bc^
1.5% coriander powder in diet	1.284	1.875^ab^	1.711^ab^
2.0% coriander powder in diet	1.319	1.868^ab^	1.704^ab^
2.5% coriander powder in diet	1.280	1.849^a^	1.676^a^
SEM	0.027	0.040	0.024

^a,b,c^Means (± standard error) within each column of dietary treatments with no common superscript differ significantly at *P* < 0.05.

**Table 7:** Hematological parameters mean (±SEM) of Ross 308 broilers fed diets or water containing the different levels of coriander (*Coriandrum sativum* L.) powder and extract at 42 days of age (mg/dL)^a,b,c,d^.

Treatment	Trait						
	Glucose	Total cholesterol	Triglycerides	Alkaline phosphatase	Uric acid	HDL cholesterol (high-density lipoproteins)	LDL cholesterol (low-density lipoproteins)
0 (control)	234.75^ab^	111.25^b^	94.50^a^	751.00^a^	3.95^ab^	73.00^a^	32.25^a^
750 ppm coriander extract in water	217.25^c^	105.25^c^	87.00^a^	677.50^a^	3.25^b^	68.75^a^	32.00^a^
1000 ppm coriander extract in water	211.25^c^	115.50^b^	107.00^a^	634.00^a^	3.27^b^	79.25^a^	30.00^a^
1250 ppm coriander extract in water	210.00^c^	113.00^b^	89.75^a^	748.80^a^	4.02^ab^	71.25^a^	34.25^a^
1.5% coriander powder in diet	231.50^b^	122.25^a^	97.75^a^	805.80^a^	4.80^a^	80.50^a^	32.00^a^
2.0% coriander powder in diet	240.75^a^	96.00^d^	98.75^a^	690.50^a^	3.62^ab^	65.25^a^	26.25^a^
2.5% coriander powder in diet	243.5^a^	109.00^bc^	109.50^a^	1060.50^a^	3.62^ab^	69.25^a^	33.25^a^

SEM	15.79	8.49	8.54	122.56	0.43	5.47	3.43

^a,b,c,d^Means (± standard error) within each column of dietary treatments with no common superscript differ significantly at *P* < 0.05.

**Table 8:** Ileum microflora mean (±SEM) of Ross 308 broilers at starter, grower, and total periods of age fed diets or water containing the different levels of coriander (*Coriandrum sativum* L.) powder and extract from 1 to 6 weeks of age (CFU/g)^a,b,c^.

Treatment	Trait			
	*Lactobacillus* bacteria	*Lactobacillus* bacteria	*Escherichia coli *	*Escherichia coli *
	(21st day of age)	(42nd day of age)	(21st day of age)	(42nd day of age)
0 (control)	7.17^a^	7.25^a^	8.76^a^	8.41^a^
750 ppm coriander extract in water	7.35^a^	7.75^a^	6.63^b^	7.23^bc^
1000 ppm coriander extract in water	7.97^a^	7.94^a^	7.31^b^	7.37^bc^
1250 ppm coriander extract in water	7.77^a^	8.19^a^	7.15^b^	7.05^c^
1.5% coriander powder in diet	7.60^a^	7.38^a^	7.31^b^	6.90^c^
2.0% coriander powder in diet	7.80^a^	7.45^a^	7.44^b^	7.36^bc^
2.5% coriander powder in diet	7.25^a^	7.33^a^	7.58^ab^	8.14^ab^

SEM	0.38	0.30	0.42	0.29

^a,b,c^Means (± standard error) within each column of dietary treatments with no common superscript differ significantly at *P* < 0.05.

**Table 9:** Immunological responses against Newcastle (ND) and avian influenza (AI) mean (±SEM) of Ross 308 broilers fed diets or water containing the different levels of coriander (*Coriandrum sativum* L.) powder and extract from 1 to 6 weeks of age (log⁡2)^a,b^.

Treatment	Trait					
	Antibody titer against Newcastle at 1st day of age	Antibody titer against Newcastle at 35th day of age	Antibody titer against Newcastle at 42nd day of age	Antibody titer against influenza at 1st day of age	Antibody titer against influenza at 35th day of age	Antibody titer against influenza at 42nd day of age
0 (control)	3.75^a^	3.75^a^	2.75^b^	3.75^a^	2.00^a^	1.25^a^
750 ppm coriander extract in water	4.00^a^	4.00^a^	5.50^a^	3.75^a^	2.25^a^	1.50^a^
1000 ppm coriander extract in water	3.75^a^	4.25^a^	5.25^a^	3.50^a^	2.50^a^	1.50^a^
1250 ppm coriander extract in water	4.00^a^	5.00^a^	5.75^a^	3.25^a^	3.25^a^	1.75^a^
1.5% coriander powder in diet	3.75^a^	4.00^a^	3.25^b^	3.50^a^	2.00^a^	1.50^a^
2.0% coriander powder in diet	3.75^a^	3.50^a^	3.00^b^	3.75^a^	2.00^a^	1.25^a^
2.5% coriander powder in diet	3.50^a^	3.50^a^	3.00^b^	3.50^a^	2.00^a^	1.25^a^

SEM	0.37	0.45	0.40	0.34	0.49	0.30

^a,b^Means (± standard error) within each column of dietary treatments with no common superscript differ significantly at *P* < 0.05.

**Table 10:** Immunological responses against infectious bronchitis (IB) and infectious bursal disease/Gumboro (IBD) mean (±SEM) of Ross 308 broilers at starter, grower, and total periods of age fed diets or water containing the different levels of coriander (*Coriandrum sativum* L.) powder and extract from 1 to 6 weeks of age (log⁡10)^a,b,c^.

Treatment	Trait			
	Antibody titer against bronchitis at 28th day of age	Antibody titer against bronchitis at 42nd day of age	Antibody titer against Gumboro at 21st day of age	Antibody titer against Gumboro at 42nd day of age
0 (control)	1527.80^a^	964.00^b^	3193.00^b^	3333.00^ab^
750 ppm coriander extract in water	1121.03^c^	283.00^c^	7427.00^a^	3675.00^a^
1000 ppm coriander extract in water	2720.00^ab^	2130.50^a^	6326.00^a^	4316.00^a^
1250 ppm coriander extract in water	3296.80^a^	2316.50^a^	7846.00^a^	4409.00^a^
1.5% coriander powder in diet	1474.30^bc^	1215.00^b^	3076.00^b^	2345.00^bc^
2.0% coriander powder in diet	808.30^c^	829.50^bc^	2960.00^b^	2529.00^bc^
2.5% coriander powder in diet	1987.30^abc^	1182.30^b^	2664.00^b^	2027.00^c^

SEM	427.75	207.19	731.10	359.83

^a,b,c^Means (± standard error) within each column of dietary treatments with no common superscript differ significantly at *P* < 0.05.

**Table 11:** Immunological responses against sheep red blood cells (SRBC) mean (±SEM) of Ross 308 broilers fed diets or water containing the different levels of coriander (*Coriandrum sativum* L.) powder and extract from 1 to 6 weeks of age (log⁡2)^a,b,c^.

Treatment	Trait					
	Total antibody against sheep red blood cell (SRBC) at 28th day of age (IgT)	Immunoglobulin G antibody against sheep red blood cell (SRBC) at 28th day of age (IgG)	Immunoglobulin M antibody against sheep red blood cell (SRBC) at 28th day of age (IgM)	Total antibody against sheep red blood cell (SRBC) at 42nd day of age (IgT)	Immunoglobulin G antibody against sheep red blood cell (SRBC) at 42nd day of age (IgG)	Immunoglobulin M antibody against sheep red blood cell (SRBC) at 42nd day of age (IgM)
0 (control)	2.37^a^	1.37^b^	1.37^b^	4.50^ab^	2.00^c^	2.37^a^
750 ppm coriander extract in water	2.87^bc^	1.50^b^	1.37^ab^	3.75^b^	2.25^bc^	1.37^b^
1000 ppm coriander extract in water	3.12^b^	1.50^b^	1.62^a^	5.37^ab^	3.37^ab^	1.87^ab^
1250 ppm coriander extract in water	4.00^a^	2.25^a^	1.75^a^	6.25^a^	4.12^a^	2.12^ab^
1.5% coriander powder in diet	2.87^bc^	1.50^b^	1.37^ab^	5.37^ab^	2.87^abc^	2.50^a^
2.0% coriander powder in diet	2.95^c^	1.37^b^	1.00^b^	4.25^b^	2.25^bc^	2.00^ab^
2.5% coriander powder in diet	2.25^c^	1.25^b^	1.00^b^	4.87^ab^	2.50^bc^	2.37^a^

SEM	0.23	0.19	0.13	0.61	0.42	0.30

^a,b,c^Means (± standard error) within each column of dietary treatments with no common superscript differ significantly at *P* < 0.05.
